# Neurobiology and Applications of Inositol in Psychiatry: A Narrative Review

**DOI:** 10.3390/cimb45020113

**Published:** 2023-02-20

**Authors:** Carmen Concerto, Cecilia Chiarenza, Antonio Di Francesco, Antimo Natale, Ivan Privitera, Alessandro Rodolico, Antonio Trovato, Andrea Aguglia, Francesco Fisicaro, Manuela Pennisi, Rita Bella, Antonino Petralia, Maria Salvina Signorelli, Giuseppe Lanza

**Affiliations:** 1 Department of Clinical and Experimental Medicine, Psychiatry Unit, University of Catania, 95123 Catania, Italy; 2 Department of Neuroscience, Rehabilitation, Ophthalmology, Genetics, Maternal and Child Health, Section of Psychiatry, University of Genoa, 16132 Genoa, Italy; 3 Istituto di Ricovero e Cura a Carattere Scientifico, Ospedale Policlinico San Martino, 16132 Genoa, Italy; 4 Department of Biomedical and Biotechnological Sciences, University of Catania, Via Santa Sofia 97, 95123 Catania, Italy; 5 Department of Medical, Surgical, and Advanced Technology, University of Catania, Via Santa Sofia 87, 95123 Catania, Italy; 6 Department of Surgery and Medical-Surgical Specialties, University of Catania, Via Santa Sofia 78, 95123 Catania, Italy; 7 Clinical Neurophysiology Research Unit, Oasi Research Institute-IRCCS, Via Conte Ruggero 73, 94018 Troina, Italy; 8 CERNUT–Research Centre for Nutraceuticals and Health Products, University of Catania, Viale A. Doria 6, 95125 Catania, Italy

**Keywords:** inositol, foods, mood disorders, psychotic disorders, anxiety disorders, neurobiology

## Abstract

Inositol is a natural sugar-like compound, commonly present in many plants and foods. It is involved in several biochemical pathways, most of them controlling vital cellular mechanisms, such as cell development, signaling and nuclear processes, metabolic and endocrine modulation, cell growth, signal transduction, etc. In this narrative review, we focused on the role of inositol in human brain physiology and pathology, with the aim of providing an update on both potential applications and current limits in its use in psychiatric disorders. Overall, imaging and biomolecular studies have shown the role of inositol levels in the pathogenesis of mood disorders. However, when administered as monotherapy or in addition to conventional drugs, inositol did not seem to influence clinical outcomes in both mood and psychotic disorders. Conversely, more encouraging results have emerged for the treatment of panic disorders. We concluded that, despite its multifaceted neurobiological activities and some positive findings, to date, data on the efficacy of inositol in the treatment of psychiatric disorders are still controversial, partly due to the heterogeneity of supporting studies. Therefore, systematic use of inositol in routine clinical practice cannot be recommended yet, although further basic and translational research should be encouraged.

## 1. Introduction

### 1.1. Background

Inositol is a natural simple sugar-like compound commonly present in many plants and foods. It belongs to the class of cyclic polyols, a group of molecules classified by the number and the position of phosphates attached to their carbon chain. Due to its chemical nature, inositol is also classified based on the bond angles between the carbon atoms and the hydroxyl group, giving rise to a wide range of stereoisomers with different biological functions [1]. As such, there are several possible isoforms of inositol in nature: muco-, neo-, scyllo-, epi-, cis-, allo-, D-chiro-, Myo- and L-chiro-inositol [2]. Apart from the biological processes regulated by its free form, inositol represents a component of complex molecules, such as inositol phosphates (Ips), phosphatidylinositol (PI), glycosyl-phosphatidylinositols, and phosphatidylinositides [3].

Inositol is involved in several biochemical pathways, most of them controlling many vital cellular mechanisms, such as cell development, signaling and nuclear processes, metabolic and endocrine modulation, cell growth, and signal transduction, among others [4]. As such, in the past few years, several studies showed anti-atherogenic, anti-oxidative, anti-inflammatory, and anti-cancer properties of this molecule [5,6,7]. Moreover, it has been reported that an alteration of inositol levels plays a pivotal role in the pathogenesis of some metabolic diseases, such as metabolic syndrome (MetS), type 2 diabetes mellitus (T2D), and polycystic ovary syndrome (PCOS), which are conditions all related to altered insulin sensitivity [8]. Recently, a potential role of inositol has also been shown for psychiatric diseases, including psychotic, mood and anxiety disorders [9,10,11,12].

In this narrative review, we aimed to present the role of inositol on human brain physiology and pathology and to provide an update on the potential applications in psychiatric disorders. To this end, we carried out a search on PubMed and PsychInfo till 6 February 2023, by using the following search string: (inositol OR “Myo-inositol” OR “D-Chiro-inositol”) AND ((unipolar OR bipolar) AND depression) OR psychosis OR schizophrenia OR Anxiety OR PTSD OR autism OR OCD OR trichotillomania OR anorexia OR “binge eating disorder” OR “substance use disorder”). The eligibility criteria were as follows: (1) studies evaluating the use of inositol in psychiatric disorders, including randomized and non-randomized studies; (2) studies written in English. The exclusion criteria were non-peer-reviewed data (e.g., abstracts or trial registry repositories) and any other publication different from research studies (e.g., review, case report, commentary, editorial). Articles describing the effects of inositol on psychiatric disorders and in in vivo studies have been included and discussed (Table 1).

### 1.2. Biochemistry and Functions

The human body synthesizes inositol at a rate of approximately 4 g per day [30]. The kidney is the organ where this process occurs the most, although other tissues (such as the brain and reproductive organs) can contribute [31]. Inositol exerts relevant functions both in its free form and as part of larger molecules, such as phosphoinositides or inositol phosphates. Moreover, different inositol-free forms can be converted to each other thanks to specific enzymes, called epimerases. Myo-inositol (mI) and D-chiro-inositol are the most common isomers present in the human body; they act as osmolytes, thus ensuring an adequate cellular defense against external and/or metabolic stressors [6,32,33].

Numerous studies on inositol metabolism converge around the biosynthesis of mI. Its biosynthetic pathway begins with the isomerization of glucose-6-phosphate into inositol-3-phosphate (Ins3p). Subsequently, Ins3p is dephosphorylated into free mI by the enzyme inositol monophosphatase-1 (IMPA-1) [34]. Myo-inositol represents one of the most important forms of phosphorus storage in many types of plants and about 1 g/day of it is provided by a normal and balanced diet [35,36]. In the gut, the absorption of mI occurs against a concentration gradient, due to the secondary coupling with a Na^+^ pump, which is non-competitively inhibited by glucose and other sugars [37]. After absorption, inositol is released into the bloodstream and then directed to the tissues. Some of these tissues own greater stocks of inositol thanks to the higher expressions of different transport systems. Accordingly, higher concentrations of free mI can be found in the male reproductive tract [38], semen [39], brain, cerebrospinal fluid (CSF), choroid plexus [40], kidney, and small intestine, whereas phospholipid-bound forms are abundant in the liver, muscles, and heart [41]. Due to its hydrophilic nature, mI at low concentration is unable to pass the blood-brain barrier and, therefore, to transport inositol into the central nervous system (CNS), doses higher than 12 g per day are necessary [42].

Different forms of inositol can also be linked to phospholipids to form PI, which are glycerophospholipids containing a variable number of the phosphate group of inositol [3]. In the form of phospholipid derivatives, they are essential components of the cells themselves as part of the membrane [43]. The synthesis of phosphoinositides involves a reaction between mI and cytidine-diphosphate-diacylglycerol, which is catalyzed by the enzyme inositol phosphatidyl transferase (PI synthase), eventually forming PI [44]. The PI obtained is subsequently phosphorylated by a series of enzymes, called phosphoinositide-kinases (PIKs), thanks to the inositol carbon atom site of phosphorylation. Through this class of enzymes, the phosphoinositides are thus synthesized and used as components of the plasma membrane. They can be the target of different enzymes which, once activated, initiate different signal transduction pathways [45].

Phosphoinositides are the most relevant inositol-containing molecules, which regulate several biological functioning, such as signal transduction, intermembrane trafficking, and actin dynamics [3]. They act by converting the phosphorylation state of inositol through a wide range of enzymes and kin phosphatases [43]. Inositol phosphates are generated exclusively by hydrolyzing the phosphoinositides or phosphating more phosphorylated forms, such as IP6. Regarding the cleavage of phosphoinositides, this activity is carried out by phospholipase (PLC). This enzyme, activated through different signals, determines the separation between the diacylglycerol (DAG) and the inositol-1,4,5-triphosphate (IP3), ref. [46] both acting as second messengers. Indeed, DAG is an activator, together with Ca^2+^, of the protein-kinase-C (PKC), being able to regulate various cellular activities in different tissues. On the other hand, IP3 can bind specific receptors on the smooth endoplasmic reticulum (IP3R), eventually leading to an increase in intracellular calcium concentrations [47].

**Figure 1:**
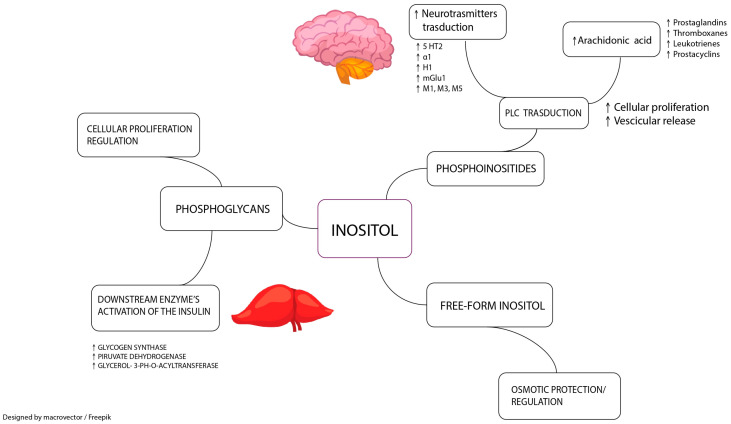
Different roles of inositol and inositol-containing molecules in human physiology. Free-form inositols, such as Myo-inositol and D-chiro-inositol, act as osmolytes to ensure adequate cellular defense against external and/or metabolic stressors. Phosphoglycans are involved in glycosyl-phosphatidylinositol/inositol phosphoglycans pathway as second messengers, regulating different cellular pathways, including insulin sensitization and cellular proliferation regulation. As phosphoinositide, inositol plays a role in phospholipase transduction, which is the signal transduction pathway of many neurotransmitter receptors. The cleavage of phosphoinositide by phospholipase activation can also release arachidonic acid, which can be subsequently converted into many inflammation mediators. M1, M3, M5: acetylcholine ionotropic receptors; H1: histamine ionotropic receptor; α1: norepinephrine ionotropic receptor; 5HT2: serotonin ionotropic receptor; mGlu1: glutamate metabotropic receptor; ↑: increase.

Finally, both mI and D-chiro-inositol are involved in the synthesis of other molecules, such as glycosyl-phosphatidylinositol (GPI) and inositol phosphoglycans (IPGs). Recently, it has been shown that these molecules act as insulin second messengers within the GPI/IPG signaling pathway. IPGs are released outside the plasma membrane under the action of insulin and thanks to a hydrolytic reaction involving GPI lipids. Both IPGs that contain mI and D-chiro-inositol can regulate insulin signal transduction by increasing its sensitivity and improving glucose metabolism [48] (Figure 1).

### 1.3. Role in Metabolic Disorders

It is well known that psychiatric conditions are associated with an increased prevalence of metabolic disorders, thus highlighting the bidirectional link between psychiatric disorders and MetS [49]. In recent years, increasing attention has been given to the role of inositol in the pathogenesis of some metabolic diseases, such as MetS [50], T2D [51], and PCOS [52], which are all conditions related to altered insulin sensitivity. Inositol, particularly mI and D-chiro-inositol, seems to act as metformin on insulin resistance, thus reducing fasting insulin, homeostasis model assessment (HOMA) index, and body mass index (BMI) [53]. In MetS, the supplementation with inositol, either alone or with α-lipoic acid, may be considered a therapeutic strategy in women since it contributes to increasing both high-density lipoprotein cholesterol (HDL-c) and HOMA index, as well as serum glucose, insulin, blood pressure, triglycerides, and total cholesterol [54,55].

Inositol was also examined in patients with glucose intolerance, given its properties as an insulin-sensitizer and second messenger, thus exerting an insulin-like effect on metabolic enzymes [54]. In type-1 diabetes (T1D), inositol was tested to replace metformin or other insulin-sensitizers often associated with insulin in T1D patients with BMI > 30. An improvement in insulin sensitivity and a significant reduction in HbA1c levels were observed, although no change was reported in BMI or insulin resistance [56].

The therapeutic role of inositol has also been investigated for T2D, a disorder characterized by insulin resistance, altered insulin release, and excessive hepatic glucose production. In T2D, inositol may be useful as a safe add-on supplement to anti-diabetic drugs, being able to decrease fasting blood glucose and HbA1c levels, whereas it did not show a significant impact on BMI, lipid profile, and blood pressure [51]. Also, pinitol, a precursor of D-chiro-inositol, seems to be effective in T2D as an add-on therapy with oral hypoglycemic drugs in lowering HbA1c, fasting glucose and, HOMA-IR, along with a reduction in patients with HbA1c > 8.0%, as well as in patients with HOMA-IR > 2.5 [57].

Additionally, inositol might modulate the endothelial dysfunction typically observed in T2D. It is known that the oral glucose tolerance test (OGTT) is the most used test to disclose an impaired glucose tolerance, although it is rather expensive, time-consuming, and can cause discomfort in patients. In this context, urinary mI levels have been used to predict T2D, showing a positive correlation with HbA1c level and fasting glucose, whereas the excretion of D-chiro-inositol did not report any correlation [58]. Indeed, high glucose concentrations inhibit the reuptake of mI in the renal tube; for this reason, diabetic patients show higher mI excretion than healthy subjects [59]. However, other studies reported a decreased D-chiro-inositol excretion in insulin-resistance patients, whereas urinary mI excretion was increased due to a competition between glucose and mI in renal tubular transport [59,60,61]. Lastly, it is well known that diabetes negatively impacts brain functions [62]. Diabetic patients often have cognitive decline, especially in terms of reduced mental speed and impaired flexibility [63]. Recurrent hypoglycemia events were considered responsible for functional brain impairment, although hyperglycemia can also significantly contribute [64].

Different strategies to prevent women from developing gestational diabetes mellitus (GDM) have been investigated, including inositol supplementation. Myo-inositol has insulin-mimetic properties implied in insulin transduction; it increases GLUT-4 translocation to the cell membrane in skeletal muscle, thus improving insulin sensitivity [65]. Inositol supplementation at 2–4 g daily, both as mI or D-chiro-inositol, in pregnant women has been associated with a lower rate of GDM and preterm delivery rate, along with a lack of effects on weight gain, hypertension, and neonatal complications (e.g., macrosomia, neonatal hypoglycemia, and respiratory distress) [66]. The efficacy of inositol in GDM seems to be dose-dependent: at lower doses (mI 1.1 mg plus D-chiro-inositol 27.6 mg) a therapeutic action on GDM is not reported [67]. Moreover, its efficacy seems to be independent of BMI, being able to reduce the incidence of GDM either for BMI < 30 [68,69,70] or >30 [71].

Lastly, PCOS is a multifactorial endocrine disorder that affects 8–13% of women of reproductive age, mainly depending on the population studied and the criteria used [72]. Given the robust link between PCOS and insulin resistance, multiple molecules acting as insulin sensitizers have been proposed to contrast the metabolic dysfunction observed in both conditions [73]. It also acts on an androgenic profile by normalizing testosterone, androstenedione, and sex hormone-binding globulin (SHBG) levels [74]. Furthermore, inositol seems to increase estradiol and decrease luteinizing hormone (LH) and leptin [75,76]. Moreover, the supplementation of inositol seems to restore spontaneous ovulation and improve oocytes’ quality [77,78,79]. However, to date, there is no conclusive consensus on the daily dose of inositol to be used. Myo-inositol seems to act well at doses of 3–4 g per day [80], while the daily dose of D-chiro-inositol should not be > 300 mg; otherwise, it might impair the oocyte quality, possibly by altering the mI/D-chiro-inositol ratio in the ovary [81]. For this reason, mI and D-chiro-inositol should be given in a “physiological plasma ratio” of 40:1 [82].

## 2. Inositol and Mood Disorders

The interest in inositol as a possible antidepressant molecule began in 1978 when Barkai and colleagues showed a reduced concentration of inositol in the CSF of patients with mood disorders [83]. Afterward, several studies measured levels of mI in different brain areas of patients with major depressive disorder (MDD) and bipolar disorder (BD), highlighting how low levels of inositol were associated with depressive symptoms, while high levels with (hypo)manic symptoms. Indeed, low levels of inositol have been found in the frontal cortex of patients affected by MDD, measured both by 1H-magnetic resonance spectroscopy (1H-MRS) and in post-mortem brain tissues [84,85,86].

The study conducted by Zheng et al. [87] showed a significant increase of mI in the left prefrontal cortex (PFC) after treatment with transcranial magnetic stimulation (TMS) in resistant depressed patients. A reduction in mI levels has also been reported for bipolar depression. Frey et al. [88] reported reduced mI concentrations in the frontal lobes of patients with MDD and bipolar depression compared to healthy controls, although these findings were significant only when the groups were matched by age. Conversely, increased mI levels were found in patients with manic and hypomanic episodes in BD. Indeed, in two studies conducted by Davanzo et al. [89,90], the presence of higher concentrations of mI in the cingulate cortex was observed in patients with BD in the manic phase compared to patients with intermittent explosive disorders and healthy controls. Consistent with these findings, a number of studies by Kato et al. reported higher concentrations of inositol in the frontal lobe of patients with manic and hypomanic episodes in BD [91,92,93]. As a whole, these findings suggest a possible role of inositol in the pathogenesis of mood disorders.

Previous studies have also proposed a possible antidepressant mechanism of inositol, which might involve the signal transduction of serotonin, one of the key neurotransmitters associated with the pathogenesis of mood disorders [94]. However, inositol does not seem to have a direct effect on synapses by inhibiting the reuptake of monoamines. Indeed, neither acute nor chronic administration of inositol has demonstrated an effect on brain levels of monoamines [95]. It has been suggested that the therapeutic activity of inositol may be related to the modulation of serotonin and/or norepinephrine receptors and to an effect on the signal transduction pathway. Indeed, from the data available in the literature, inositol acts as a precursor of the inositol phosphate-phosphoinositide (IPP) cycle, which is the source of two-second messengers, i.e., IP3 and DAG. The IPP cycle and its derived second messengers are involved in several receptor systems, including noradrenergic (α-1), serotonergic (5-HT_2A_ and 5-HT_2C_), cholinergic (muscarinic), and dopaminergic (D1) receptors [96,97].

A study conducted by Einat et al. [98] investigated the effect of inositol on an experimental model of depression applied to laboratory rats. In particular, the authors evaluated the effects of the administration of inositol alone and in association with inhibitors of both the serotonergic and noradrenergic systems, to investigate which neurotransmitter pathway was involved in determining the clinical effect of inositol. As a result, the effects of inositol were blocked by inhibitors of the serotonergic system only (through the 5-HT_2A_/5-HT_2C_ receptors), although the authors could not exclude other pathways in the effects of inositol, such as the involvement of the hypothalamic-pituitary-adrenal (HPA) axis [98].

Two studies evaluated the use of inositol as an adjunct treatment to selective serotonin reuptake inhibitors (SSRIs) in patients with MDD. Both of them did not find any difference in the reduction of depressive symptoms, assessed by the Hamilton Depression Rating Scale (HDRS), in patients treated with inositol versus placebo [13,14]. Regarding the treatment of BD, inositol did not show any significant clinical improvement. Indeed, in both the studies by Chengappa et al. [15] and Evins et al. [99], inositol was added to treatment with mood stabilizers, showing no statistically significant difference compared to the control group. Finally, in a study conducted by Levine et al. [11] in both MDD and BD patients in the depressive phase, 12 g of inositol was administered for 4 weeks, showing a significant reduction in HDRS scores in the group treated with inositol compared to the placebo group. However, according to the meta-analyses conducted by Mukai et al. [100] and Schefft et al. [101], inositol did not show a clear antidepressant effect. Therefore, although neuroimaging and biomolecular studies showed a potential role of inositol in the pathogenesis of mood disorders, clinical studies are still controversial.

The potential efficacy of inositol has also been studied in the treatment of premenstrual dysphoric disorders (PMDD), in which depressive symptoms appear regularly and only during the second half of the menstrual cycle, and then disappear with or immediately after menstruation. Two clinical trials, conducted by Nemets et al. [17] and by Gianfranco and al. [16], showed a trend towards the efficacy of inositol monotherapy compared to placebo. The meta-analysis by Mukai et al. [100] showed a tendency of inositol to counteract depressive symptoms in the context of PMDD.

Lastly, inositol has been proposed not only as a possible antidepressant agent but also as a molecule capable of counteracting the side effects of mood stabilizers, such as lithium and sodium valproate, which are common drugs used for the treatment of mood disorders, in particular BD [102]. Previous studies suggested that lithium and valproate may reduce the concentrations of inositol within the CNS. It was proposed that lithium and sodium valproate may induce alterations in the PI cycle, thus lowering mI concentrations [91,103]. However, the so-called “inositol depletion hypothesis” remains the most controversial hypothesis for the mechanism of action of lithium and sodium valproate in BD [103]. In this context, several studies have shown how the administration of these drugs changes the levels of inositol in the brain. For example, Davanzo et al. [89] found a significant reduction in brain inositol levels after lithium intake in patients with BD during a (hypo)manic episode. Another study, conducted by Sharma et al. [104], showed similar results. Sodium valproate was found to decrease inositol biosynthesis [105]. On the contrary, Patel et al. [106] investigated differences in mI concentrations after lithium administration, showing no significant change in mI levels after 42 weeks of treatment in patients with bipolar depression. Thus, the accuracy of the inositol hypothesis as an explanation for the clinical efficacy of lithium and sodium valproate remains uncertain [107]. Although the reduction in the concentration of inositol in the CNS may play a role in determining the effects of these drugs, the reduction of peripheral inositol levels observed in other tissues may explain some of the side effects, resulting from the intake of lithium and sodium valproate. For instance, a reduction in inositol concentration in the kidney could account for the polyuria/polydipsia developed by treated patients [108], as well as for the hypothyroidism and weight gain, which are common side effects of these molecules [109]. For these reasons, reducing or moderating these side effects through inositol supplementation has been recently evaluated [110].

In conclusion, inositol as monotherapy or in addition to conventional antidepressant drugs did not seem to influence clinical outcomes in mood disorders.

## 3. Inositol and Psychotic Disorders

It has been demonstrated that astrocytes might be implied in different tasks, playing a core role in the pathogenesis of schizophrenia, e.g., supporting myelinization, removing glutamate from intrasynaptic space, and contributing to maintaining synaptic integrity and the redox brain balance [111]. Myo-inositol is more present in astroglia than in the other brain cells [112] and, for this reason, the detection of changing levels of mI through MRS may provide information about the astroglia status [113], e.g., a lower level of mI may signal insufficient astroglial activity. In this scenario, a pioneering study in a small sample of patients with schizophrenia demonstrated that oral inositol significantly increased its CSF level by almost 70%, thus suggesting therapeutic applications [18].

In patients with schizophrenia at an early and untreated stage, it was demonstrated a reduction in the concentration of mI in the anterior cingulate cortex (AAC), while after 6 months of treatment, the levels of mI appeared restored, showing as mI could be a good marker of symptoms regression in an early stage of schizophrenia, along with a reduction of the positive and negative syndrome scale (PANSS) total score [114]. Kubota et al. showed that mI levels in the thalamus were significantly increased after treatment, although this occurred in small group sizes, suggesting that antipsychotics should exert beneficial effects on glial integrity in patients affected by schizophrenia [115].

The increase of mI in MRS may occur when there is a brain injury, in astrogliosis, or in augmented astrocytic proliferation and demyelination [116]. Subjects at clinical or genetic high risk for psychosis usually show higher levels of mI in the dorsolateral prefrontal cortex (DLPFC) [117], whereas subjects with genetic risks (siblings of psychiatric patients) were detected with higher levels of mI, also in the medial prefrontal cortex [118], thus suggesting that these changes might be a sign of biological vulnerability.

Based on these findings, which underline the potential implication of mI in the pathogenesis of psychosis, inositol supplementation was assessed as a treatment for schizophrenia in three studies, conducted between 1993 and 1994 by Levine et al. [19,20,119] and then reported in a meta-analysis [120]. In the first crossover randomized controlled trial (RCT) [19], inositol supplementation was given to patients with chronic schizophrenia at a dose of 6 g per day for 10 days in add-on to the antipsychotic therapy. No improvement in the brief psychiatric rating scale (BPRS) score was reported compared to the control group. The study was replicated in the same year [119], extending the treatment duration from 10 days to 4 weeks, although without differences in the results. In the last RCT conducted by Levine et al. [20] 12 g of inositol was given to subjects with schizophrenia every day for four weeks, producing no significant results. The following year (1995), based on the evidence that inositol metabolism was involved in the second messenger system for several muscarinic cholinergic receptors and that cholinergic agonists were reported to ameliorate the memory deficit induced by electro-convulsive therapy (ECT), 6 g of inositol was administered daily in a crossover double-blind study for 5 days before the 5th or 6th ECT in a series of patients, including 5 with schizophrenia): no effect was found on post-ECT cognitive impairment [29].

Overall, inositol was not superior to placebo in reducing psychotic symptoms [121].

## 4. Anxiety Disorders

Studies on the effectiveness of inositol in anxiety disorders began in 1995, after the publication of findings using inositol as a treatment for depression. In the first RCT conducted by Benjamin et al., inositol was more effective than a placebo in reducing the number of panic attacks experienced during the four-week trial. On the other hand, these clinical positive results were not related to a significant improvement in the HDRS or in the Hamilton anxiety rating scale (HARS) [26].

In 1996, Kaplan et al. [25] conducted an RCT on 13 patients with post-traumatic stress disorder (PTSD), who received 12 g of inositol supplementation per day for four weeks. No improvements in the core symptoms of PTSD (such as avoidance and intrusion) were observed in the inositol group, although a reduction of depressive symptoms after treatment was noted in a subgroup of five people. Similarly, a single dose of 20 g of inositol did not attenuate the meta-chlorophenylpiperazine-induced anxiety, mydriasis, and endocrine effects in panic disorder, although it had some acute effects during the evening before the challenge [122].

In 2001, Palatnik et al. conducted a crossover RCT on 21 patients with panic disorder, comparing the use of inositol and fluvoxamine. The study reported comparable efficacy between inositol and fluvoxamine in reducing the HARS total score, the Marks-Matthews Fear Questionnaire (for the evaluation of agoraphobia), and the clinical global impressions (CGI) scale. Of note, a higher incidence of fatigue and nausea was noted in the fluvoxamine group only. This data, although scarce, may suggest the use of inositol in panic disorder, especially in subjects poorly tolerating SSRIs [10]. Accordingly, preliminary results from more recent reviews on pharmacological interventions in panic disorder concluded that inositol has antipanic properties and may be an effective compound in the treatment of panic disorder [123,124]. Recently, Zulfarina et al. [125] have proposed the use of inositol in the Malaysian guidelines for the treatment of panic disorders, alongside other conventional treatments or even as a monotherapy drug.

Overall, encouraging results seem to emerge for inositol in panic disorders, likely through its peculiar second messenger characteristics, which are different from the transmitter-receptor mechanism of SSRIs used for this disorder. This might be implied also for the behavioral effects with adaptation occurring after chronic administration of inositol [126,127] and the evidence that the anxiolytic effect depends on the baseline level of anxiety [127]. However, these findings have been demonstrated in animal models only and, therefore, randomized controlled studies in large samples of patients are needed.

## 5. Other Psychiatric Disorders

Studies in obsessive-compulsive disorder (OCD) were discordant, with some indicating efficacy against a placebo in a small group of patients [22,23], while others showed no statistical significance [21,22] Moreover, the meta-analysis conducted by Mukai et. [100] found no relevant difference against placebo. However, it should be noted that chronic inositol administration increased striatal dopamine D2 receptors but did not modify dexamphetamine-induced motor behavior. As such, D2 receptor upregulation may play an important role in the behavioral effects of inositol in OCD, although the role of the serotonin 5HT2 receptor in this response is still debated [128].

The use of inositol for the treatment of trichotillomania did not show any efficacy in a single 10-week trial on 38 patients [24].

Finally, the therapeutic role of inositol has been examined for bulimia nervosa and binge eating disorder. The crossover trial conducted by Gelber et al. [28] evaluated the efficacy of 18 g of inositol per day in 12 patients over 12 weeks. The results showed an improvement in the CGI scales, the Visual Analog Scale, and the Eating Disorders Inventory, corroborating the parallelism with SSRIs [28].

Lastly, in a crossover trial by Levine et al. [27], inositol supplementation (200 mg/kg body weight) did not demonstrate any efficacy compared to placebo in a small sample of patients with autism spectrum disorders (ASD). Therefore, as recently recommended by a systematic review on the effect of dietary supplements on clinical aspects of ASD, caution is needed when interpreting these results [129].

## 6. Conclusions

Over the last few years, several studies have suggested the efficacy of inositol in different metabolic disorders. Given that many metabolic pathways are also involved in the pathogenesis and course of psychiatric disorders, inositol might represent the trait-d-union between these clinical conditions. However, despite its multifaceted neurobiological activities, to date, literature evidence on the efficacy of inositol in the treatment of psychiatric disorders is still controversial, partly due to the heterogeneity of supporting studies. Moreover, the design of the current study is subject to several limitations due to the exploratory nature of the present work. Therefore, systematic use of inositol in routine clinical practice cannot be recommended yet, although further basic and translational research should be encouraged.

## Figures and Tables

**Table 1:** Summary of the studies included.

Authors, Year Study Design Type of Disorder Treatment Duration Follow-Up Characteristics of the Population Studied	Interventions	Measures	Study Aim (s) Efficacy
**Mood Disorders**			
Levine et al. 1995a [11] Design: Randomized Clinical Trial (RCT) Treatment duration: 4 weeks Participants: Major Depressive Disorder (MDD), Bipolar Disorder (BD) (28) Male/female: 12/16 Mean age (range): inositol group 63.7 (35–80) Control group 50.5 (20–71) Diagnosis: MDD	Intervention group: Inositol 12 g/day Control group: Glucose	Hamilton Depression Rating Scale (HDRS)	Study aim: to evaluate the efficacy of administration of inositol for the treatment of depression. Results: Inositol group showed an improvement in the HDRS scores.
Nemets et al. 1999 [13] Design: RCT Treatment duration: 4 weeks Participants: MDD (36) Male/female: 14/22 Mean age (range): Selective Serotonin Reuptake Inhibitors (SSRI) + Inositol group 49.5 ± 11 SSRI + control group 51.5 ± 11 Diagnosis: MDD in patients who have failed to respond to SSRI	Intervention group: Inositol 12 g/day + SSRI Control group: Glucose + SSRI	HDRS	Study aim: to evaluate the efficacy of administration of inositol in augmentation with SSRI for the treatment of depression. Results: inositol group did not improve depression in SSRI treatment failures.
Levine et al. 1999 [14] Design: RCT Treatment duration: 4 weeks Participants: MDD (27) Male/female: 8/19 Mean age (range): SSRI + Inositol group 45.9 ± 5 SSRI + control group 49.6 ± 5 Diagnosis: MDD	Intervention group: Inositol 12 g/day + SSRI Control group: placebo + SSRI	HDRS	Study aim: to evaluate the clinical response to the addition of inositol to SSRIs in the treatment of depression. Results: inositol group did not show any significant improvement in depression.
Chengappa et al. 2000 [15] Design: RCT Treatment duration: 6 weeks Participants: BD depression (22) Male/female: 8/14 Mean age (range): inositol group 38 ± 8 Control group: 47 ± 13 Diagnosis: BD depression	Intervention group: inositol 12 g/day + mood disorders drugs Control group: placebo + mood disorders drugs	HDRS; Clinical Global Improvement (CGI-I); Montgomery–Asberg Depression Rating Scale (MADRS); Udvalg for Kliniske Undersøgelser Side Effects Scale (UKU); Young Mania Rating Scale (YMRS)	Study aim: to evaluate the inositol’s potential efficacy and safety in BD. Results: 6 patients of the inositol group showed a decrease in the baseline HDRS score and in the CGI-I scale compared to 3 subjects assigned to placebo. On the MADRS, 8 patients of the inositol group showed decreased scores compared to 4 subjects assigned to the placebo group.
**Premenstrual Dysphoric Disorder (PMDD)**			
Gianfranco et al. 2011 [16] Design: RCT Treatment duration: 6 menstrual cycles Participants: PMDD (71) Male/female: 71 females Mean age (range): 18–45 years Diagnosis: PMDD	Intervention group: myo-inositol powder 12 g OR myo-inositol capsules soft gel 3.6 g/day Control group: powder or soft gel capsule placebo	Penn Daily Symptoms Records scale (DSR), HDRS, Clinical Global Impression-Severity of Illness scale (CGI-S)	Study aim: to evaluate the effect of myo-inositol on the treatment of PMDD Results: the inositol group showed significant improvement in DSR, HDRS and CGI-S scales.
Nemets et al. 2002 [17] Design: RCT Treatment duration: 6 menstrual cycles Participants: PMDD (12) Male/female: 12 females Mean age (range): 35.9 ± 5 (30–43) Diagnosis: PMDD	Intervention group: myo-inositol 12 g/day Control group: Placebo	Tension Analog Scale, Irritability Analog, Sadness Analog, Headache Analog, Bloating Analog, Breast Tenderness, HDRS, CGI-S, Self-rating for premenstrual tension syndrome (PMTS)	Study aim: to evaluate the inositol’s efficacy in PMDD Results: inositol was not found to be superior to placebo in any of the scales used.
**Psychotic Disorders**			
Levine et al. 1993a [18] Design: RCT crossover Treatment duration: 4 weeks Participants: 10 Male/female: 6/4 Mean age (range): 36.8 (32–48) Diagnosis: schizophrenia	Intervention group: myo-Inositol 6 g/day + antipsychotic therapy Control group: Mannitol + antipsychotic therapy	Brief Psychiatric Rating Scale (BPRS)	Study aim: to evaluate the efficacy of the administration of inositol for the treatment of schizophrenia. Results: no overall effect of 6 g daily inositol on total symptoms scores was found.
Levine et al. 1993b [19] Design: RCT crossover Treatment duration: 10 days Participants: 11 Male/female: 7/4 Mean age (range): 53.2 (33–60) Diagnosis: schizophrenia	Intervention group: Inositol 6 g/day + antipsychotic therapy Control group: Lactose + antipsychotic therapy	BPRS	Study aim: to evaluate the efficacy of the administration of inositol for the treatment of schizophrenia. Results: no overall effect of 6 g daily inositol on total symptoms scores was found.
Levine et al. 1994 [20] Design: RCT crossover Treatment duration: 4 weeks Participants: 12 Male/female: 4/8 Mean age (range): 44.7 (26–63) Diagnosis: schizophrenia	Intervention group: Inositol 12 g/day + antipsychotic therapy Control group: dextrose + antipsychotic therapy	Positive and Negative Syndrome Scale (PANSS)	Study aim: to evaluate the efficacy of inositol supplements and the level of inositol in cerebrospinal fluid of schizophrenic patients. Results: no overall effect of 12 g daily inositol on total symptoms scores was found.
**Obsessive-Compulsive Disorder (OCD)**			
Fux et al. 1996 [21] Design: RCT crossover Treatment duration: 6 weeks Participants: OCD with lack of response to previous treatments with SSRIs or clomipramine or with reported side effects to previous treatment (13) Male/female: 5/8 Mean age (range): 33.7 years (23–56) Diagnosis: OCD	Intervention group: Inositol 18 g/day Control group: glucose	Yale-Brown Obsessive-Compulsive Scale (Y-BOCS), HDRS, Hamilton Anxiety Rating Scale (HARS)	Study aim: to evaluate the efficacy of inositol in the treatment of OCD. Results: no statistical significance was observed between inositol and placebo.
Fux et al. 1999 [22] Design: RCT double-blind crossover Treatment duration: 6 weeks Participants: OCD (10) with inadequate response to serotonin reuptake inhibitors (SRI) therapy Male/female: 2/8 Mean age (SD): 30.3 (9) Diagnosis: OCD	Intervention group: Inositol 18 g/day as an add-on to SRI treatment Control group: glucose	Y-BOCS, HDRS, HARS	Study aim: to confirm the efficacy of inositol in OCD Results: there were no significant differences between inositol and placebo treatment.
Seedat et al. 1999 [23] Design: open-label trial Treatment duration: 6 weeks Participants: OCD (10) with lack of adequate response to SRI treatments Male/female: 3/7 Mean age (range): 33.3 ± 14.2 years Diagnosis: OCD	Intervention group: inositol 18 g/day as add-on to SRI treatments	Y-BOCS, CGI-S, MADRS	Study aim: to evaluate the efficacy of inositol in patients with lack of adequate response to SRI treatments. Results: there were significant improvements in CGI-S and Y-BOCS scales in the inositol group but not in MADRS scores.
**Trichotillomania**			
Leppink et al. 2016 [24] Design: RCT Treatment duration: 10 weeks Participants: Tri (38) Male/female: 3/35 Mean age (range): 28.9 ± 11.4 Diagnosis: trichotillomania	Intervention group: inositol 6 g/day, after two weeks 12 g/day, after two weeks 18 g/day Control group: placebo powder	Massachusetts General Hospital Hair Pulling Scale, NIMH Trichotillomania Severity Scale, CGI-S, Sheehan Disability Scale (SDS), HARS, HDRS, Quality of life Inventory (QoLI)	Study aim: to investigate the efficacy of inositol in trichotillomania Results: no improvement in symptoms was reported in the inositol group.
**Post-traumatic Stress Disorder (PTSD)**			
Kaplan et al. 1996 [25] Design: RCT Treatment duration: 4 weeks Participants: PTSD (13) Male/female: 8/5 Mean age (range): 39.7 (25–56) Diagnosis: PTSD	Intervention group: inositol 12 g/day Control group: placebo powder (glucose)	Impact of Event Scale (IES), Symptom Check List (SCL-90), HDRS and HARS	Study aim: to assess the efficacy of inositol in PTSD Results: no improvement in PTSD core symptoms (avoidance and intrusion) was reported in the inositol group.
**Anxiety Disorders**			
Benjamin et al. 1995 [26] Design: RCT Treatment duration: 4 weeks Participants: 21 Male/female: 9/12 Mean age (SD): 35.8 (7) Diagnosis: panic disorder with or without agoraphobia	Intervention group: Inositol 12 g/day and lorazepam in case of anxiety Control group: mannitol or glucose powder and lorazepam in case of anxiety	Marks-Matthews Phobia Scale, HARS, HDRS	Study aim: to evaluate the efficacy of inositol in anxiety and panic disorder. Results: frequency and severity of panic attacks, phobia scores and panic scores were significantly improved in the inositol group in comparison to the control group.
Palatnik et al. 2001 [10] Design: RCT crossover Treatment duration: 1 month Participants: 21 patients Male/female: 9/12 Mean age (SD): 39.2 (11) Diagnosis: panic disorder with or without agoraphobia	Intervention group: Inositol 18 g/day Control group: fluvoxamine up to 150 mg/day	Summation of entries in a daily panic diary, HARS, CGI-S, HDRS, Marks-Matthews Fear Questionnaire	Study aim: to evaluate the efficacy of inositol in comparison to fluvoxamine in the treatment of panic disorder. Results: inositol and fluvoxamine had approximately the same efficacy at reducing HARS, phobia and CGI-S scores and inositol was slightly more effective than fluvoxamine at reducing the number of panic attacks with fewer collateral effects in the inositol group.
**Autism Spectrum Disorder (ASD)**			
Levine et al. 1997 [27] Design: RCT crossover Treatment duration: 4 weeks Participants: autistic children (10) Male/female: 9/1 Mean age (SD): 5.6 ± 3.2 Diagnosis: autism	Intervention group: myo-inositol 200 mg/kg/day, in one case in add-on with carbamazepine for epilepsy Control group: dextrose	Childhood Autism Rating Scale (CARS), CGI-S, Conners Parent-Teacher Questionnaire (CONNERS)	Study aim: to evaluate the efficacy of inositol in autism disorder. Results: no benefit showed in children with autism of inositol.
**Bulimia Nervosa (BN) and Binge Eating Disorders (BED)**			
Gelber et al. 2001 [28] Design: RCT crossover Treatment duration: 6 weeks Participants: BN, BED (12) Male/female: 1/11 Mean age (range): 24 (20–39) Diagnosis: BN, BED	Intervention group: inositol 18 g/day Control group: grape sugar	Eating Attitude Test (EAT), Visual Analog Scale of severity of binge eating (VAS-B), CGI-S, Eating Disorders Inventory (EDI), HDRS, HARS, 14-item side effects inventory.	Study aim: to assess the efficacy of inositol in BN and BED. Results: inositol group showed significant improvements in CGI-S and VAS-B, with a borderline significant effect on the EDI in comparison to the control group. No statistical difference was reported for EAT.
**Others**			
Levine et al. 1995b [29] Design: RCT crossover Treatment duration: 5 days before the 5th or 6th RCT Participants: in treatment with ECT (15) Male/female: 5/10 Mean age (range): 49 (27–72) Diagnosis: MDD, Schizoaffective disorder depressed, neuroleptic non-responsive schizophrenia	Intervention group: inositol 6 gr/day Control group: dextrose	HDRS, BPRS, Cognitive function tests	Study aim: to assess whether inositol might enhance cholinergic function and reverse ECT-induced memory impairment Results: no effects were found on post-ECT cognitive impairment.

## Data Availability

Not applicable.
